# Graphene-Enriched Acrylic Paint to Protect Unheated and Heat-Treated Wood Species Against *Coniophora puteana*

**DOI:** 10.3390/polym18121462

**Published:** 2026-06-11

**Authors:** Hamid R. Taghiyari, Elham Nadali, Antonio Pizzi, Roya Majidi, Jakub Kawalerczyk, Ioanna A. Papadopoulou, Olaf Schmidt, Antonios N. Papadopoulos

**Affiliations:** 1 Wood Science and Technology Department, Faculty of Civil Engineering, Shahid Rajaee Teacher Training University, Tehran 16788-15811, Iran; 2 Natural Resources Faculty, Semnan University, Semnan 35131-19111, Iran; e.nadali@yahoo.com; 3 Laboratoire d’Etude et Recherche sur le Materiau Bois (LERMAB), University of Lorraine, 27 Rue Philippe Seguin, 88000 Epinal, France; antonio.pizzi@univ-lorraine.fr; 4 Department of Physics, Faculty of Sciences, Shahid Rajaee Teacher Training University, Tehran 16788-15811, Iran; r.majidi@sru.ac.ir; 5 Department of Mechanical Wood Technology, Poznań University of Life Sciences, 60637 Poznań, Poland; jakub.kawalerczyk@up.poznan.pl; 6 Department of Chemistry, Aristotle University of Thessaloniki, 54124 Thessaloniki, Greece; papad.ioanna@chem.auth.gr; 7 Wood Biology, Institute of Wood Science, University of Hamburg, Leuschnerstr. 91d, 21031 Hamburg, Germany; 8 Department of Natural Environment and Climate Resilience, Democritus University of Thrace, 66100 Drama, Greece

**Keywords:** biological durability, compression strength parallel to grain, graphene, heat treatment, mass loss, wood-decay fungi

## Abstract

Graphene was added to acrylic paint to be coated on two sets of unheated and heat-treated specimens of three commercial wood species (namely beech, poplar, and spruce) to protect against *Coniophora puteana*. Heat treatment was carried out at the mild temperature of 185 °C for four hours in a laboratory oven and under atmospheric pressure. Each of the two sets were divided into three sub-groups of uncoated (control), coated with plain paint, and coated with graphene-enriched paint to be exposed to the fungus. Results showed that coating of specimens with the plain acrylic paint significantly protected all three wood species against the fungus. Still, reinforcing effect of graphene resulted in an even higher degree of protection, and it slightly increased compression strength compared to grain as well. Heat treatment also improved biological resistance in all three wood species, which is seen in the drastic decrease of the mass losses. It also increased compression strength as a result of hornification and thermal alterations of cell-wall polymers. It was concluded that graphene-added acrylic paint can be recommended as an easy and available superficial protecting method to significantly protect both hard- and softwoods against *C. puteana*.

## 1. Introduction

As an organic material, wood is susceptible to many organisms, including wood-decay fungi, insects and termites, and marine borers, not to mention the vulnerability of wood towards fire and high temperatures. Apart from the above, its dimension changes as a result of contact with water, or when it remains in areas with high humidity for a long time. Still, it has unique and remarkable properties, making it a preferable material for many applications, ranging from structural projects to artistic works of art. Therefore, researchers have carried out many studies to find ways to overcome its inherent drawbacks.

In terms of the susceptibility of wood to wood-decay fungi, many preservatives (mostly chemical formulations which are toxic) have already been studied and presented to the industry [1,2]. These chemicals have brought up environmental concerns especially over the past decades in the world, though there is no doubt about their effectiveness in practice. Moreover, chemical modification methods, like acetylation and furfurylation, also have their own environmental problems [3,4,5,6,7,8,9,10]. Therefore, researchers tried to include environmentally-friendly methods. In this connection, one of the most acceptable methods is heat treatment which not only improves the biological resistance of wood against wood-decay fungi but also improves its dimensional stability [11,12,13,14].

Heat treatment can be carried out under different mediums, like hot atmospheric air, hot water, boiling water, and some inert gases such as nitrogen. Heat treatment can be done under mild temperatures of lower than 190 °C, or under higher temperatures, usually at or above 200 °C, technically called thermal modification. The duration and regime of the heating temperatures can also significantly affect the results. During heat treatment, chemical structure and composition of wood polymers (mainly cellulose, hemicellulose, and lignin) are changed in a way that the enzymes secreted by the wood-decay fungi cannot digest the modified wood [15]. Although heat treatment can definitely improve biological resistance in wood species, it can substantially weaken mechanical properties in the modified wood as well, due to thermal degradation of wood polymers. Therefore, researchers mostly intend to find an optimum heat treatment regime (degree and duration of heating temperature) in which the maximum biological resistance is achieved along with the minimum loss in mechanical properties. Based on this, one of the objectives in this study was to investigate the extent of effectiveness of heat treatment at the mild temperature of 185 °C on the biological resistance of three commercial wood species (namely beech, poplar, and spruce), popular in Iran’s market.

As discussed above, heat treatment is commercially considered a well-known modification method for solid wood species to improve both biological durability and dimensional stability as well. However, it requires apparatuses that might not be available in every wood workshop, and it involves expenses that may not be commercial for every application. Therefore, it may be helpful to present a fast and easy way for protection of wood that can be used everywhere, and with the lowest possible expenses involved. In this connection, superficial wood coatings and finishes are considered an easy method to protect the wood substrate. However, most of the existing wood coatings need to be improved to provide a long-term protection.

Graphene has attracted significant attention as a multifunctional additive in polymeric coatings due to its exceptional mechanical strength, high surface area, and reported antimicrobial properties. Its incorporation into coatings can act as a physical barrier, enhance matrix integrity, and potentially restrict microbial growth, including fungi, on coated surfaces. Recent studies have demonstrated that graphene-based additives can improve the antifungal performance of coatings applied to wood and other polymer substrates. For instance, Li et al. [16] reported that graphene oxide-modified polyurethane coatings reduced mold colonization on wood surfaces. Yaragalla et al. [17] found that graphene nanoplatelets incorporated into acrylic coatings enhanced barrier properties and inhibited fungal growth. Moya et al. [18] observed improved water resistance and antifungal activity in graphene-reinforced polymer coatings. Based on these findings, the present study explored the incorporation of graphene into an acrylic paint as a potentially effective, low-cost, and easy-to-apply strategy to enhance the fungal resistance of both unheated and heat-treated wood species. Despite the unique improving impact of graphene on many properties of polymer matrices (including mechanical, thermal, fire, and antimicrobial), its uniform dispersion is considered a great challenge for researchers, due to strong van der Waals interactions with the polymers, causing agglomeration [16]. Therefore, various techniques have been tested, like ultrasonication, functionalization of graphene surfaces, the use of different surfactants [19,20]. In the present study, dispersing was done using a magnetic stirrer to keep the costs as low as possible for future application of the technique by the commercial sector.

Therefore, graphene was added to an acrylic paint, as a low-cost and common type of coating with an increasing popularity. It was hypothesized that the reinforcing effect of graphene would significantly improve the protecting property of the acrylic paint. Moreover, graphene was reported to have antibiotic property that can hinder wood-decay fungi mycelium to pass through to get to wood substrates. The antibiotic property of graphene acts based on three mechanisms, including: (i) physical deterioration of the attacking bio-organisms, (ii) causing oxidative damages to the organisms, (iii) forming a physical barrier impregnable for nutrients and moisture to pass through [17,21]. Based on the experimental design used in the present research study, the results can be used for both the academic and commercial sectors.

## 2. Materials and Methods

### 2.1. Specimen Preparation

Three commercial wood species were selected based on their maximum popularity and availability on the market. From the hardwoods, beech (*Fagus orientalis* Lipsky) and poplar (*Populus nigra* Lipsky) were chosen to provide two hardwoods with different density categories. From the softwoods, spruce (*Pice aabies* L.H. Karst.) was chosen to be in the same density range of poplar, for comparison purposes. Based on the market information, the beech lumbers purchased were originally grown in the northern province of Gillan (Iran), and the lumbers of poplar were cultivated in the western province of Kurdistan (Iran). Densities of beech and poplar lumbers were 0.64 and 0.43 g⋅cm^−3^, respectively. Spruce is not a native tree in Iran, and the lumbers, with an average density of 0.44 g⋅cm^−3^, were imported from Tver province (Russia).

**Figure 1:**
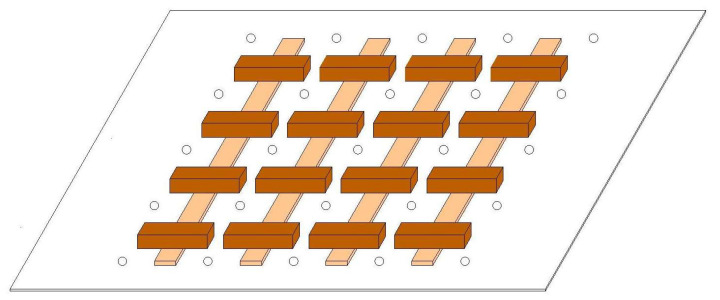
Specimens put on wood strips and randomly arranged on a metal tray to be heat-treated at 185 °C for four hours in a laboratory oven.

Totally, 90 specimens were selected, thirty for each of the three wood species. The specimens were carefully checked to be free of any checks, knots, any fungal or insect defects, or any other possible visible defects. The specimens were all in longitudinal direction, and the size was 20 mm × 20 mm × 50 mm. The specimens were randomly divided into two sub-groups of “unheated” and “heat-treated”. Heat treatment was carried out in a laboratory oven at 185 °C for four hours. The specimens were randomly placed on 3-mm thickness wood strips to avoid their direct contact with the metal tray in the oven which might cause over-heating (Figure 1). Once heat-treated, they were conditioned in a climate chamber (25 ± 2 °C, 45 ± 3% relative humidity) for a month before being coated by either plain or graphene-added acrylic paint.

The fifteen specimens in each sub-group were divided into three groups of five specimens each, namely the control (uncoated) group, the painted group coated with plain acrylic paint, and the graphene-painted group coated with graphene-enriched acrylic paint. Acrylic paint was selected because of its growing popularity for both outdoor and indoor wood applications [22]. The paint was produced by Alvan Paint and Resin Production Co. Ltd. (Tehran, Iran), with product code ALCO-6510. According to the manufacturer’s technical data sheet, the paint contained 37 ± 1% solids. Both plain acrylic and graphene-enriched acrylic paints were applied by brush in two coats, with a 24 h interval between successive applications. All six surfaces of each specimen, including the two end-grain surfaces, were completely coated to ensure uniform coverage and to minimize moisture and fungal ingress through uncoated wood surfaces. The two-coat application resulted in an average dry-film thickness of 210 ± 10 μm. The paint thickness was measured by a destructive coating thickness gauge (model 121/4); it was produced by Elcometer Co. (Manchester, UK). The average consumptions of the plain and graphene-enriched acrylic paints were measured to be 2.70 g and 3.79 g, respectively. Due to variations in wood porosity and paint absorption, local thicknesses may differ slightly; the reported value represents an average for all specimens within each treatment group. Graphene was incorporated into the acrylic paint at a concentration of 12% based on the wet weight of the paint. This concentration was chosen based on the results of a preliminary study to check the optimum concentration of graphene. Moreover, the similarity of graphene concentration to a previous study on a mineral (namely, wollastonite) dispersed in an acrylic paint would provide a basis for comparison for the commercial sector [23]. An HS-860 model magnetic stirrer was used, produced by Alfa Lab Co. (Tehran, Iran). After complete drying of the coatings, the specimens were conditioned along with the control specimens under controlled conditions (25 ± 2 °C and 45 ± 2% relative humidity) for two additional months. Prior to fungal incubation, the average moisture content of the specimens was measured to be 9 ± 0.5%.

Graphene was incorporated into the acrylic paint until visually homogeneous before application. While the addition of graphene may have altered paint properties such as viscosity, drying time, or surface wettability, these parameters were not measured in the present study. Therefore, any influence of graphene on these properties is speculative and requires further investigation.

### 2.2. Production of Graphene as a Paint-Reinforcer

Graphene used as a paint-reinforcer was produced via electrochemical exfoliation of graphite, following the procedure reported in [24,25]. A platinum cathode (0.5 × 10 cm^2^) and a graphite foil anode (2 × 10 cm^2^) were placed 2.7 cm apart in an aqueous electrolyte solution of NiCl_2_·6H_2_O (0.05 M, 98%, Merck KGaA, Darmstadt, Germany) and subjected to a constant voltage of 10 V. HCl was periodically used to clean the platinum electrode every 20 min to prevent excessive graphene accumulation on the cathode. Exfoliated graphene was collected by vacuum filtration [26,27,28]. The resulting graphene was thoroughly washed with deionized water to remove any residual Ni ions. While no characterization of the graphene produced in the present study was performed, the method has been shown in previous work to yield few-layer graphene sheets with minimal defects and negligible nickel contamination [24,25]. The collected graphene was subsequently used to prepare graphene-enriched acrylic paints.

### 2.3. Fungal Incubation

Once prepared and cut to size, the specimens were exposed to a brown-rot fungus, namely *Coniophora puteana* (Schumach.) P. Karst. The isolate 167 of this fungus was identified by rDNA-ITS sequencing and derived from the collection prepared by Olaf Schmidt (University of Hamburg, Hamburg, Germany) [29]. Before inoculation, the specimens were autoclaved (121 ± 3 °C, 20 min). Preserving jars (500 mL) with 100 mL malt agar were prepared; the malt agar contained (2% malt extract, 1.5% agar, and Oxoid). The specimens were incubated in the jars when the top surface of the agar was totally overgrown by *C. puteana* mycelium. As the duration of fungal exposure was long and in order to prevent moisture loss, the cap of each jar was sealed by one layer of parafilm. The incubation was carried out in a conditioned chamber (Wood Laboratory at the University of Hamburg, Hamburg, Germany). Following four months of incubation, the specimens were taken out of the jars to be delicately cleaned of the overgrown mycelia on their surfaces. Once cleaned without distorting the wood substrate, all were weighed to be put in a laboratory oven. The specimens were heated at 103 °C for 24 h, followed by weighing them to calculate the mass loss for each and every exposed specimen.

### 2.4. Measurement of the Mechanical Property

Based on the limitation of specimen size for fungal exposure, compression strength parallel to grain was decided to be measured in the specimens exposed to *C. puteana*. Separate sets of specimens were also prepared to measure the values without being exposed to the fungus so that the difference in the values provides a criterion to estimate the impact of fungal exposure on at least one mechanical property. Compression strength tests were measured according to the specifications described in DIN 52-185 standard. Tests were done in a model Zwick/Roell Z050 universal testing machine at Thünen Institute of Wood Research (Hamburg-Bergedorf, Germany). The load cell capacity was 50 kN and the loading speed was determined to be 0.8 mm⋅min^−1^. Having measured the maximum forces by the universal testing machine, the values for the compression strength parallel to grain for each specimen were calculated according to Equation (1).

(1) Pc||  = FmaxA (Nmm2)

In Equation (1), $P_{c||}$ represents the compression strength parallel to grain value which is in N/mm^2^; *F*_max_ is the maximum force in N as registered by the universal testing machine; and *A* indicates the cross section of the specimen (mm^2^) along which the force was applied to them.

### 2.5. SEM Imaging

For preparation of the specimens, they were first cut to size 10 mm × 10 mm × 2 mm; the directions were according to the target surface for imaging. Then, a thin layer was cut from the target surface, using a razor blade. The cut layers were fixed on separate aluminum stubs, using carbon paste, to be later sputter-coated with a thin layer of gold. Finally, scanning electron microscopy was carried out using an FEI Quanta 250 FESEM (field-emission) (FESEM, FEI Company, Hillsboro, OR, USA).

### 2.6. Statistical Analysis

Statistical analysis was carried out based on differences in variances of treatments (ANOVA) by SPSS software (version 18, 2010). A 95% level of confidence was considered to ascertain significant differences (*p* < 0.05), followed by Duncan multiple range test to determine similar and dissimilar groupings among treatments. Minitab software (16.2.2, 2010) was utilized to design fitted-line, contour, and surface plots, as visual presentation of trends between the measured properties. In order to find out how treatments of each wood species were grouped based on all the studied properties, hierarchical cluster analyse was performed by SPSS, based on the mean raw data, using Ward’s method for linkage among the groups.

## 3. Results and Discussions

### 3.1. Mass Loss

**Figure 2:**
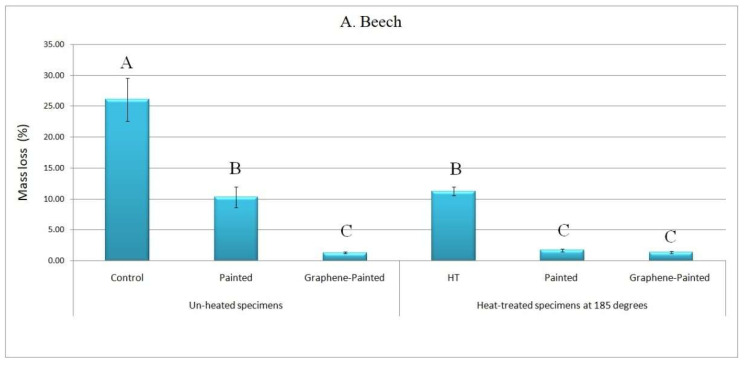

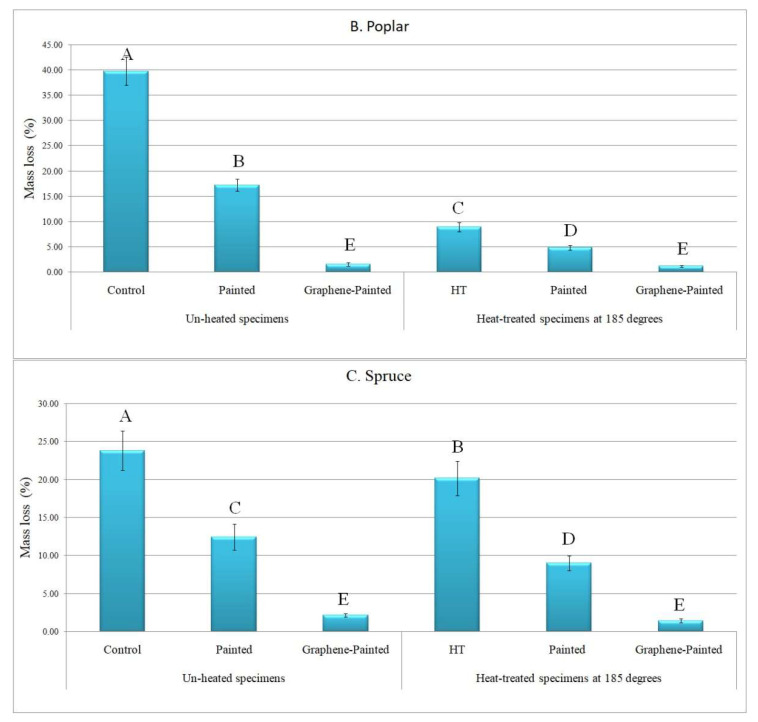
Mass losses (%) in the unheated and heat-treated solid wood species of beech (**A**), poplar (**B**), and spruce (**C**), after being exposed to *Coniophora puteana* brown-rot fungus for four months (Painted = coated specimens with plain acrylic paint; Graphene-Painted = coated specimens with graphene-added acrylic paint; HT = heat-treated specimens) (Letters on top of each column represent the Duncan multiple range groupings at 95% level of confidence).

Comparison of the weights of the specimens before and after being exposed to *C. puteana* revealed that the values were statistically different, at the 5% significance level. The highest mass losses (ML) were found in the control (unheated unpainted) specimens in all three species (Figure 2A–C). ML values were 26.1%, 39.8%, and 23.8% in the control specimens of beech, poplar, and spruce specimens, respectively. Heat treatment demonstrated a significant decreasing effect on mass losses in all species. ML values in the heat-treated specimens (unpainted) decreased by 56%, 77%, and 15% in comparison to those ML values in beech, poplar, and spruce control specimens, respectively.

Coating of the unheated specimens with the plain acrylic paint illustrated a statistically significant (*p* < 0.05) protection against the fungus. ML values demonstrated 60%, 56%, and 47% decreases in comparison to the ML values occurred in the unheated beech, poplar, and spruce specimens, respectively (Figure 2A–C). Addition of graphene to the acrylic paint resulted in an even greater protection of the unheated specimens in all three species. In the heat-treated specimens, addition of graphene had a significant protecting effect only in poplar and spruce specimens (Figure 2B,C); heat-treated beech specimens demonstrated no significant alteration in ML as a result of the addition of graphene (Figure 2A).

### 3.2. Compression Strength Parallel to Grain

Compression strength parallel to the grain was calculated using the nominal cross-sectional dimensions of the specimens. Although fungal degradation may have locally reduced the effective load-bearing area, the specimens generally maintained their overall geometry, and the original dimensions were used consistently across all treatment groups to facilitate comparative evaluation of residual mechanical performance.

**Figure 3:**
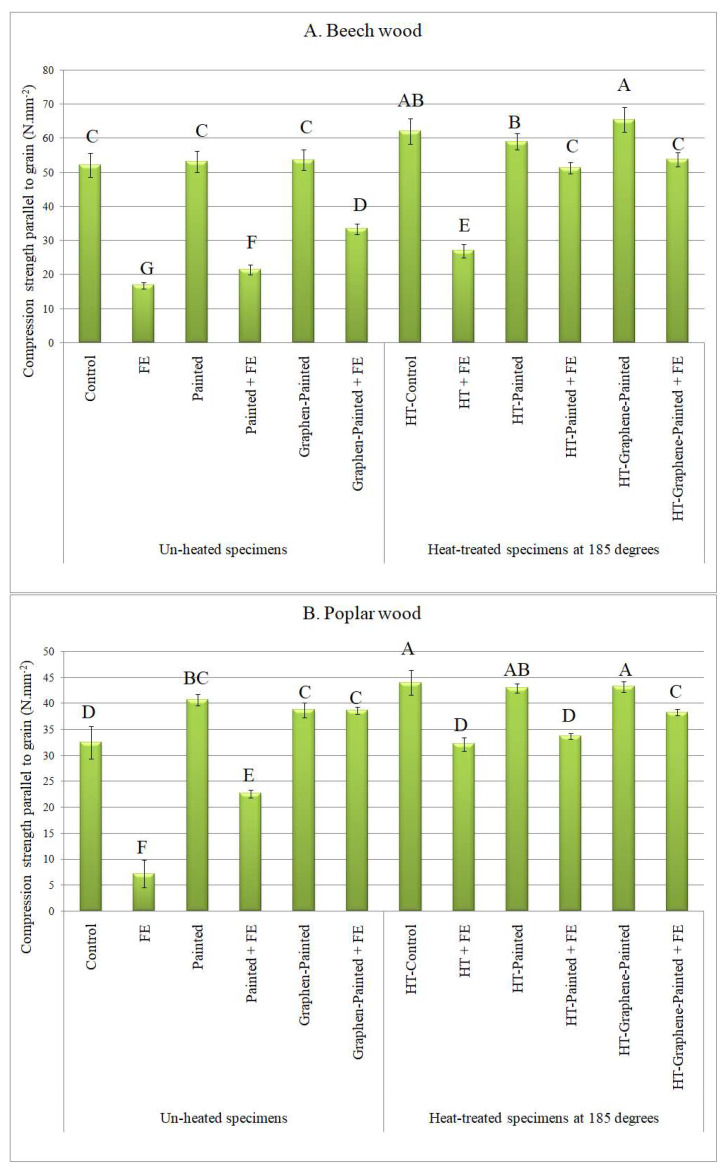

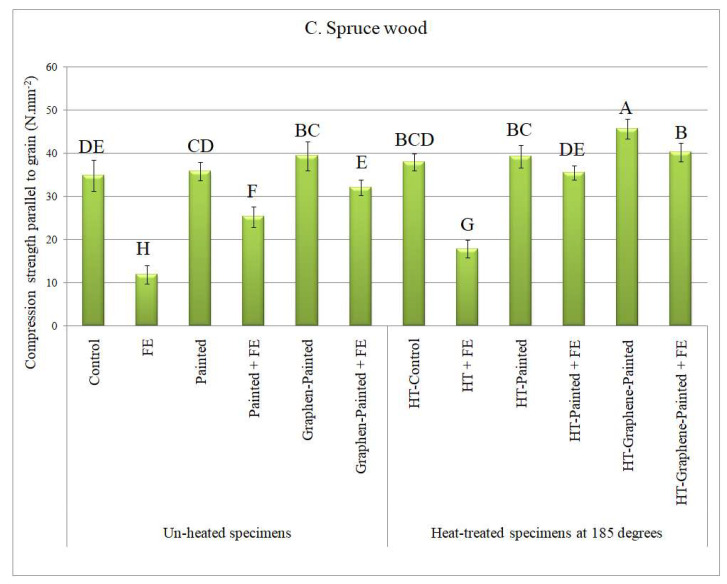
Compression strength parallel to grain (N⋅mm^−2^) in the unheated and heat-treated wood species of beech (**A**) and poplar (**B**) as hardwoods, and spruce (**C**) as a softwood, exposed to *Coniophora puteana* in glass jars for four months (FE = specimens exposed to the fungus for four months; Painted = coated specimens with plain acrylic paint; Graphene-Painted = coated specimens with graphene-added acrylic paint; HT = heat-treated spruce specimens) (Letters on the columns represent the statistical groupings, at 95% level of confidence).

Exposure of the control (uncoated, unheated) specimens to *C. puteana* for four months resulted in statistically significant decreases (*p* < 0.05) in compression strength parallel to grain in all three species (Figure 3A–C). Beech specimens generally demonstrated higher compression strengths in comparison to their poplar and spruce counterparts. The lowest and highest compression strengths parallel to grain were observed in the fungi-exposed control poplar specimens (7.21 N⋅mm^−2^) and the unexposed heat-treated beech specimens coated with graphene-added acrylic paint (65.45 N⋅mm^−2^), respectively. Exposure of the control specimens to the fungus resulted in 67%, 77%, and 65% decreases in the beech, poplar, and spruce specimens, respectively.

**Figure 4:**
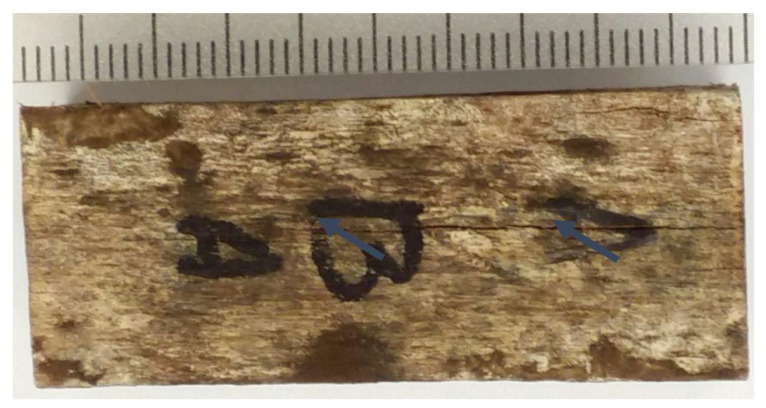
Splitting mode of failure (↓) along the length of a heat-treated fungi-exposed beech specimen coated with plain acrylic paint; the coating is almost completely destroyed after four months of exposure to *Coniophora puteana*. (Each of the scaled bars represents one millimeter).

Heat treatment had an increasing impact on the compression strength values of unexposed specimens in all three studied species. Exposure of heat-treated specimens to the fungus caused drastic decreases in compression strength of beech and spruce, though the extent of decreases as a result of exposure was lower in comparison to the unheated specimens. In poplar specimens, the decrease was not as steep, though still statistically significant. Exposure of the heat-treated (uncoated) specimens to the fungus resulted in 56%, 26%, and 52% decreases in the beech, poplar, and spruce specimens, respectively. The majority of the heat-treated specimens demonstrated a splitting mode of failure along the longitudinal direction of the specimens (Figure 4).

Coating of the specimens, regardless of being either unheated or heat-treated, with the plain acrylic paint significantly ameliorated the negative effect of the fungal exposure on compression strength values, though the values still demonstrated significant differences from those of the unexposed specimens. Addition of graphene to the paint showed a significant degree of protection in both unheated and heat-treated specimens of all three species. In the unheated poplar specimens, the protecting effect was so high that the exposed specimens coated with graphene-added paint were categorized in the same statistical grouping as their unexposed counterparts.

## 4. Discussion

**Figure 5:**
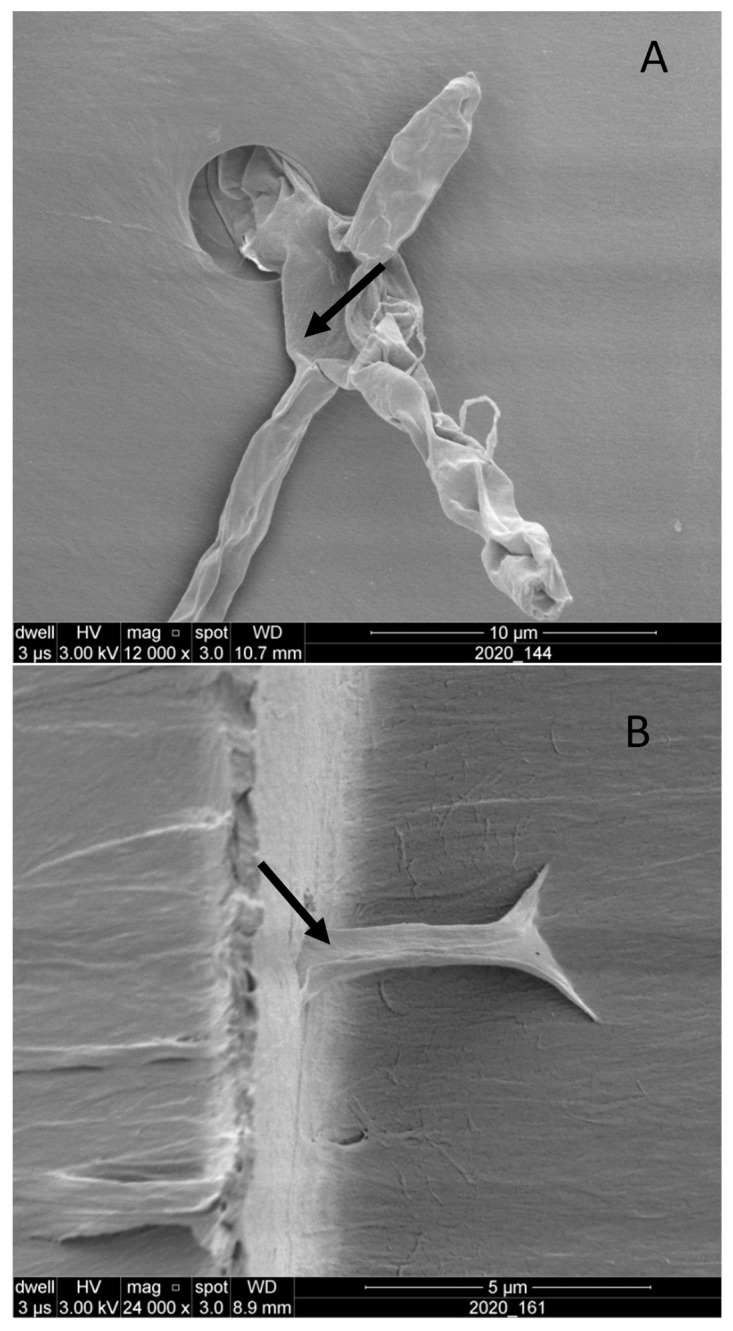
SEM micro-images of mycelium of Coniophora puteana fungus (↓) extending through a cell-wall pit (**A**) and opening (**B**) to the adjacent cell.

**Figure 6:**
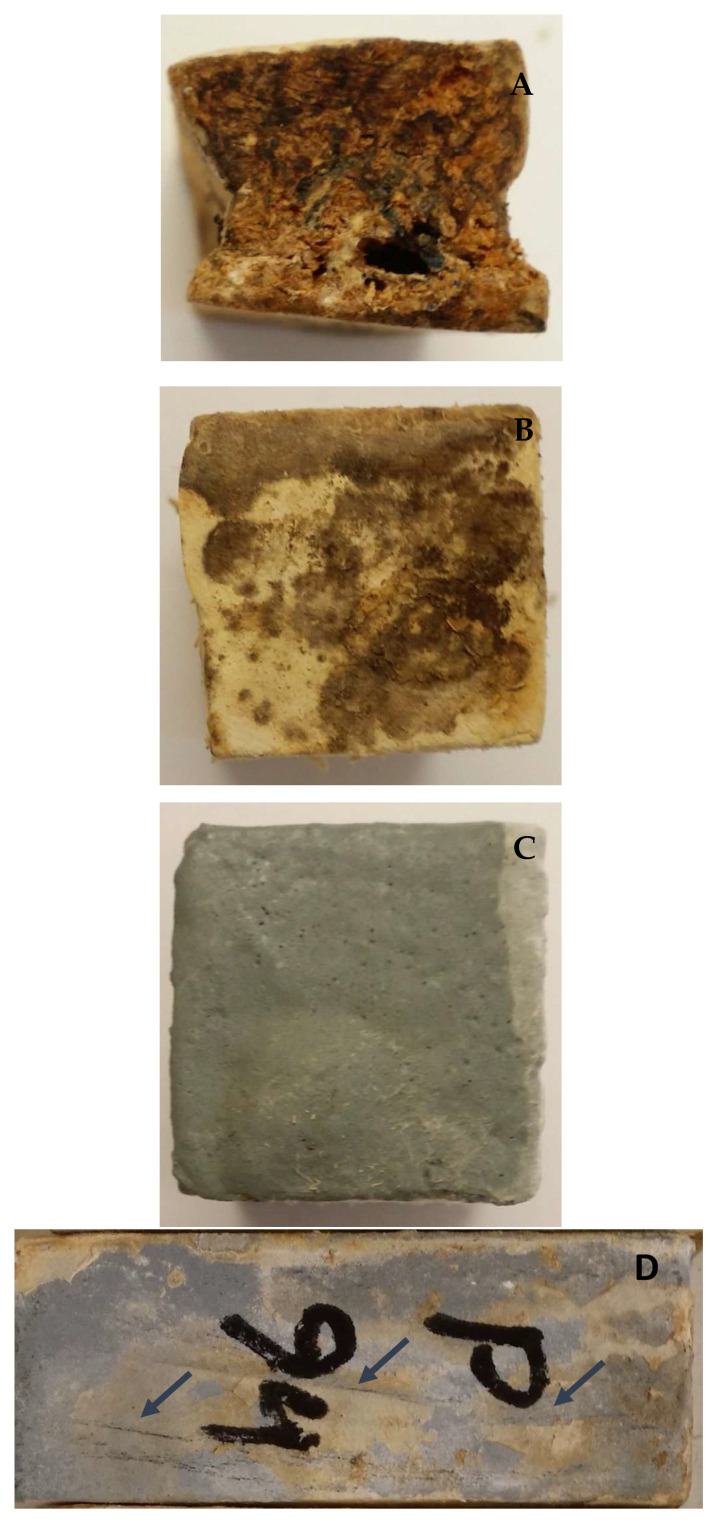
Cross-sectional view of the specimens after having been exposed to *Coniophora puteana* for four months; (**A**) the control (unheated unpainted) specimen showing complete deterioration of the wood, (**B**) the coated specimen showing destroyed layer of the plain acrylic paint, (**C**) the specimen showing the intact layer of graphene-added acrylic paint, and (**D**) scattered cracks (↓) on the longitudinal side of a specimen coated with graphene-added acrylic paint.

**Figure 7:**
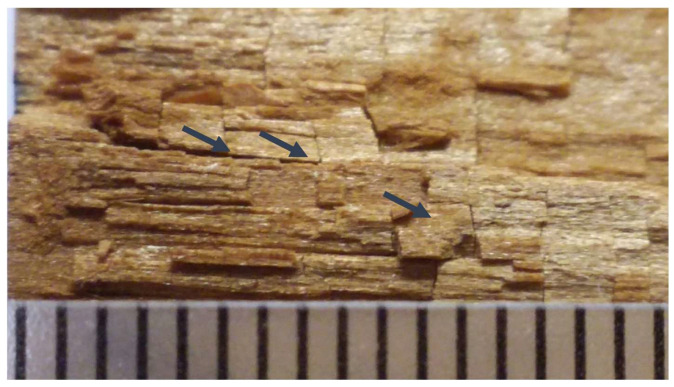
Severe fungal decay caused by *Coniophora puteana* demonstrating cubic deterioration (↓) which is typical of brown-rot fungi. (Each of the scaled bars below the photo represents one millimeter).

Scanning electron microscopy illustrated mycelium of the fungus penetrated from one cell to the adjacent one through ultra-small pits and openings (Figure 5A,B). The drastic decay and mass losses in the uncoated specimens (Figure 6A and Figure 7), regardless of being either unheated or heat-treated, were attributed to the lack of any obstacles to block the penetration of *C. puteana* fungus, resulting in an easy progression of the fungus mycelium deep into the uncoated specimens even at the early stages of the incubation. In this connection, coating of the unheated specimens with the plain acrylic paint resulted in 60%, 56%, and 47% decreases in ML in beech, poplar, and spruce species, respectively. Based on this, it can be concluded that even a mere coating of solid wood with plain acrylic paint can significantly and effectively protect the wood substrate against *C. puteana* and the consequent damages. Moreover, comparison of plain and graphene-added acrylic paints clearly demonstrated that graphene could significantly improve the extent of protection. Visual examination of the fungi-exposed specimens revealed that the plain paint layer was severely damaged, and in some parts of the specimens was completely destroyed, after four months of exposure (Figure 6B). On the other hand, the layer of paint was mostly intact on the cross-sectional view of the specimens coated with graphene-added paint (Figure 6C), though scattered cracks could be observed on the longitudinal sides (Figure 6D). This implied that the reinforcing effect of graphene gave a high integrity to the paint layer even after four months of exposure to the fungus in damp conditions. As corroborating evidence to the reinforcing effect of graphene on the acrylic paint, the increased compression strength values in the unexposed specimens coated with graphene-added paint can be noticed; in some cases, the increased values were statistically significant in comparison to their counterpart specimens coated with the plain acrylic paint (Figure 3A–C). Although not all treatments demonstrated an increased or a significant increased value, compression strengths were generally higher in the specimens coated with graphene-added acrylic paint. For instance, compression strengths of the unexposed unheated and heat-treated spruce specimens were 10% and 16% higher, respectively, than their counterpart specimens coated with plain paint. Therefore, it was concluded that the graphene acted as a reinforcer in the acrylic paint, improving its integrity over time and in damp conditions, so that the graphene-added paint layer efficiently acted as a physical block against the penetration of *C. puteana* mycelium. In addition to acting as a reinforcer in the acrylic paint, the generation of reactive oxygen by graphene (causing oxidative damage to bio-organisms) was also partially influential in restricting the growth and penetration of the fungal mycelium into the wood substrates [17,21]. Based on the above findings, further studies can provide a more comprehensive outlook on the optimum concentration of graphene to protect against various wood-decay fungi. Moreover, reinforcing effects of other materials that have been used as paint additives and reinforcers should also be studied to be compared with graphene to ultimately determine the most effective reinforcer.

As it was expected and based on previous studies [12,13,15], heat treatment significantly improved biological resistance of the control (uncoated) wood species (Figure 2A–C). Measurement of ML in the unheated and heat-treated specimens with no coating demonstrated significantly lower values in the heat-treated specimens in comparison to their unheated counterparts; the highest improvement was observed in the poplar specimens (77%), followed by beech (56%) and spruce (15%). In this connection, structural changes in the chemical composition and cell-wall polymers caused by thermal degradation have repeatedly been reported by researchers [11,12,13,14]. These changes prevent the main wood polymers (mainly cellulose, hemicellulose, and lignin) from being easily broken by fungi enzymes, resulting in the significant decreases in ML values. Compression strengths parallel to grain provided corroborating evidence for the chemical changes and structural micro-rearrangement that occurred as a result of the heat treatment. Among the unexposed specimens, the heat-treated demonstrated significantly higher compression strengths compared to their unheated counterparts, in all three species (Figure 3A–C). The increased compression strength was attributed to the formation of new cross-linking bonds that occurs during a process called glass transition of lignin [12,30,31]. In this connection, it was reported that some covalent bonds are broken between lignin and hemicellulose as a result of heat treatment. Consequently, some fragments are formed in lignin that have lower molecular weight but higher reactivity [11,31,32]. As a result of condensation of lignin and repolymerization of these fragments, lignin is rearranged based on new cross-linking bonds that are formed between lignin and hemicellulose. The rearranged lignin improves the overall strength of the heat-treated solid wood species.

In addition to the above-mentioned glass transition of lignin, a simultaneous process happens during heat treatment, called irreversible hydrogen bonding, which is also partially influential in the increased compression strengths in the present study [14]. During this process, wood polymer molecular chains more easily move to form a new arrangement in cell-wall structure [33,34]; at the same time, water molecules irreversibly leave wood, leading to a phenomenon referred to as hornification. As a result of the rearrangement of cell-wall polymers that occurs along with the loss of a portion of water molecules, some “irreversible hydrogen bonds” are formed among the wood polymers [14,35,36,37]. These two phenomena, namely glass transition of lignin and irreversible hydrogen bonding during hornification, caused the increase in compression strength parallel to grain in the heat-treated specimens.

**Figure 8:**
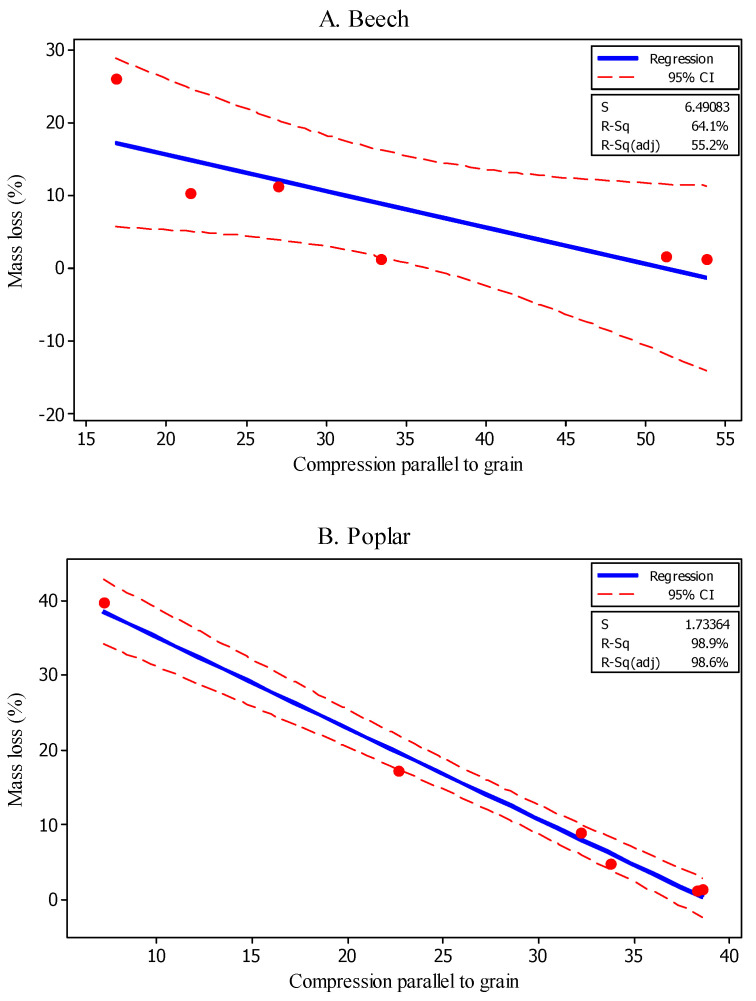

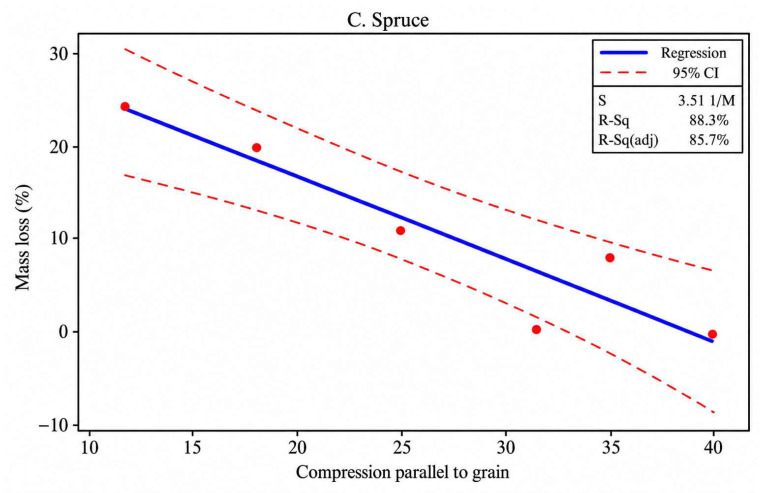
Fitted-line plot between compression strength parallel to grain values versus mass losses caused by *Coniophora puteana* for four months in beech (**A**), poplar (**B**), and spruce (**C**) specimens.

**Figure 9:**
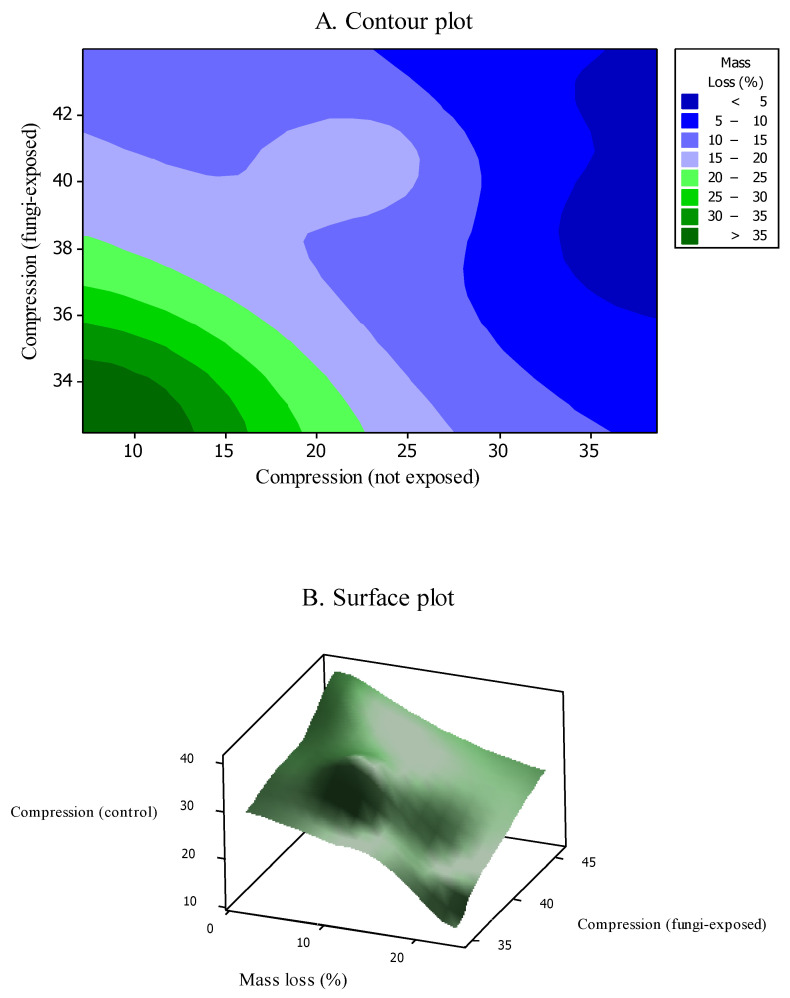
Contour (**A**) and surface (**B**) plots of poplar and spruce wood species, respectively, based on different properties measured (including mass losses, compression strengths parallel to grain before and after being exposed to *Coniophora puteana* for four months).

The correlation between the mass losses and the compression strengths (after being exposed to the fungus) was high and statistically significant in all three species, though differences in R-squared values were obvious between them (Figure 8A–C). Considering the fluctuations in mass losses and compression strengths caused by the two variables of heat treatment and coating (by either plain or graphene-added acrylic paints), it can be concluded that while both variables had statistically significant effects on the results, their interaction differed on each wood species. In this connection, an obvious general trend was shown by contour and surface plots, revealing the same impacts of heat treatment and coating in all three species, though the slight discrepancies in both plots indicated the impact of variables varied from one species to another (Figure 9).

**Figure 10:**
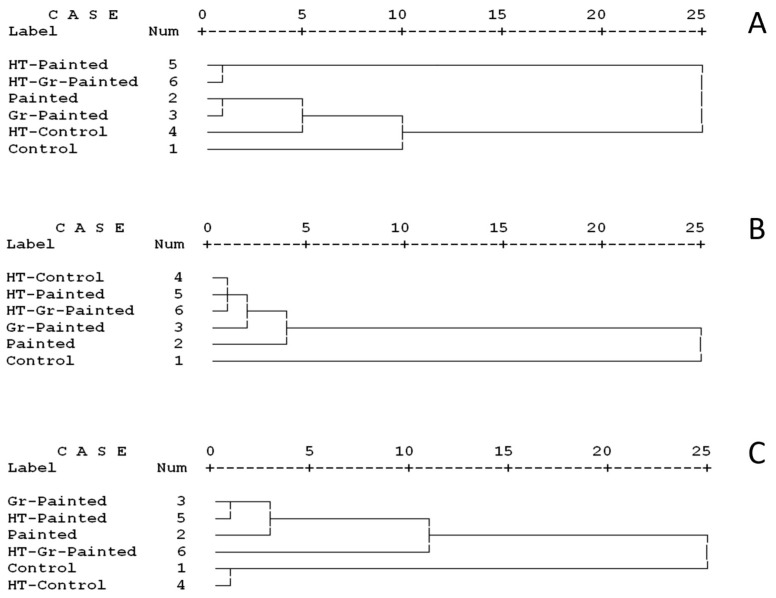
Separate cluster analyses of the treatments of each of the three wood species, including beech (**A**), poplar (**B**), and spruce (**C**); the cluster analyses were conducted based on the properties measured: mass losses and compression strength parallel to grain before and after being exposed to *Coniophora puteana* (Gr = graphene-added paint; HT = heat-treated specimens).

Separate cluster analyses were carried out for each of the wood species based on the three properties measured in this study, including: mass losses, compression strengths parallel to grain before and after being exposed to *C. puteana* fungus. The analyses demonstrated that the control treatment (unheated, unpainted specimens) in all species were clustered quite remotely from the other treatments (Figure 10A–C). In beech specimens, the heat-treated specimens were positioned between the control and coated treatments (either with plain or graphene-added paints) (Figure 10A). However, heat-treated specimens in poplar were remotely clustered relative to the control specimens (Figure 10B). Cluster analysis of spruce showed that heat-treated specimens (unpainted) were closely clustered with the control specimens (Figure 10C). This clearly indicated that heat treatment at 185 °C had the least protecting effect in spruce wood, based on the mass loss and compression strength values of control and heat-treated spruce specimens. On the other hand, heat treatment had a quite significant impact on the overall results in poplar specimens; the impact can clearly be observed in the drastic differences in the mass losses and compression strength values of the control and heat-treated poplar specimens (Figure 2B and Figure 3B). As to the beech wood, the impact of heat treatment was between the two other species.

In terms of the effect of the coating of specimens with acrylic paint, the painted specimens were remotely clustered from the control specimens in all three wood species. This clearly illustrated the significant protecting effect of a mere layer of the plain acrylic paint against the decaying effect of *C. puteana*. However, it can be deduced that the paint was more effective in poplar and spruce as the joining points in the cluster analyses of these two species were far more remotely positioned, based on the scale-bar above the cluster analyses. On the other hand, the impact of the paint in the unheated beech specimens was lower, though statistically significant.

Addition of graphene to the acrylic paint seemed to have no significant effect in either unheated or heat-treated beech specimens as the treatments were closely clustered together (Figure 10A). However, addition of graphene resulted in a significant impact on the overall properties of the unheated poplar and spruce specimens. In terms of the heat-treated specimens of poplar and spruce, addition of graphene had a significant protecting effect on spruce specimens, but it showed no significant impact on poplar specimens.

Based on the above discussion, it was concluded that heat treatment at 185 °C significantly improved biological durability of all three species against *C. puteana*, though the extent of protection was the least in spruce specimens. In terms of the effect of coating, a mere layer of the plain acrylic paint can significantly protect the solid wood substrates, regardless of the species. However, as wood can be located in damp areas and under severe conditions, addition of graphene to the paint can even significantly improve the integrity of the paint layer so that it remaines intact for effective protection of the wood substrate over long period of times. Graphene is hypothesized to enhance the coating performance by improving matrix integrity and acting as a physical barrier, which may help restrict fungal colonization. Additionally, the generation of reactive oxygen species (ROS) by graphene could contribute to antifungal activity. However, these mechanisms were not directly measured in the present study, and further investigation including coating cross-sectional analysis, graphene distribution, and water uptake or vapor permeability measurements is needed to confirm these effects.

The improved fungal resistance observed in the graphene-enriched coatings may be associated with the presence of graphene within the acrylic matrix. Previous studies have reported that graphene can enhance the mechanical and barrier properties of polymeric coatings. However, no direct physico-mechanical characterization of the coating films was performed in the present study. Therefore, the contribution of graphene to coating integrity and mechanical reinforcement should be regarded as a plausible explanation rather than a demonstrated mechanism.

Simultaneous application of heat treatment and coating with graphene-added paint can be recommended to the industry as a reliable protection method against *C. puteana*. Still, further field studies need to be carried out to ensure that the graphene-added acrylic paint can provide a practical protection of large-scale wood bodies in service.

The results of this study indicated that graphene-enriched acrylic coatings can enhance the resistance of wood against fungal colonization. However, the performance of these coatings under other environmental and mechanical conditions, such as UV exposure, abrasion, cyclic humidity, freeze-thaw cycles, dust, or salt mist, was not investigated. Therefore, while the present findings suggest potential benefits, further studies are needed before the coatings can be recommended for broader practical applications.

## 5. Conclusions

There are many techniques and methods to protect wood against wood-decay fungi. However, many of the methods require devices and machinery that are not available in remote areas. Therefore, finding an effective method which is easy and available everywhere will be helpful in preserving wood. The results of the present study demonstrated that graphene can be added to an acrylic paint as a reinforcer of the paint, improving its strength and integrity. Its oxidizing property against bio-organisms was also partially effective in hindering the growth of wood-decay fungi of *Coniophora puteana*. This provided a practical protecting method against the fungus that can be easily applied on wood everywhere with the minimal equipment and technology. Moreover, heat treatment at the mild temperature of 185 °C also significantly improved biological durability in wood species as a result of chemical alteration of wood cell-wall polymers. Based on the results, it was concluded that applying graphene-added acrylic paint on heat-treated wood not only improved compression strength parallel to grain as a mechanical property, but it also provided a satisfactory protection against the wood-decay fungus *C. puteana*. Still, further studies can give a better outlook on the optimum graphene content to be mixed in acrylic or other paint types.

## Data Availability

Data are contained and presented within the article.
